# Intraoperative indeterminate frozen-section margin leading to an unintended R1 resection in perihilar cholangiocarcinoma: a case report and decision-making framework

**DOI:** 10.3389/fsurg.2026.1899125

**Published:** 2026-08-04

**Authors:** Xiaojing Li, Jia Wang, Liye Liu, Ying Wei, Yanan Wang, Jiaxin Sun

**Affiliations:** 1 Department of Pathology, The Affiliated Hospital of Inner Mongolia Medical University, Inner Mongolia, China; 2 Inner Mongolia Autonomous Region People's Hospital, Inner Mongolia, China; 3 Department of Hepatobiliary Surgery, The Affiliated Hospital of Inner Mongolia Medical University, Inner Mongolia, China; 4 Central Laboratory, The Affiliated Hospital of Inner Mongolia Medical University, Inner Mongolia, China

**Keywords:** frozen section, indeterminate margin, perihilar cholangiocarcinoma, surgical decision-making, unintended r1 resection

## Abstract

Intraoperative frozen-section (FS) margin assessment is essential for guiding precise resection in perihilar cholangiocarcinoma (pCCA). However, freezing artifacts, superficial sampling, drainage-related reactive epithelial changes, and subtle cytologic atypia in well-differentiated adenocarcinoma can make intraoperative interpretation difficult. We report a 70-year-old woman with Bismuth-Corlette type II pCCA who underwent radical extrahepatic bile duct resection after PTBD-mediated biliary decompression. Intraoperative FS of the distal bile duct margin showed atypical glands of undetermined nature, while proximal margins were negative. Because additional caudal resection would probably have required combined pancreaticoduodenectomy in an elderly patient recovering from severe obstructive jaundice, the surgical team avoided extended resection after real-time surgical-pathological discussion. Permanent sections and immunohistochemistry subsequently confirmed well-differentiated adenocarcinoma involving the distal margin, perineural invasion, and regional lymph-node metastasis, consistent with an unintended R1 resection. This case illustrates that an indeterminate FS margin should be regarded as a high-risk gray-zone finding rather than a safe negative result. We propose a three-tier margin risk-stratification framework in which a “presumed positive” principle triggers repeat oriented sampling, intraoperative multidisciplinary consultation, and individualized balancing of oncologic radicality against physiological reserve. When aggressive resection is contraindicated, meticulous documentation, early adjuvant therapy, and rigorous long-term surveillance are essential.

## Introduction

Perihilar cholangiocarcinoma (pCCA) is an aggressive biliary malignancy arising from the hilar bile duct epithelium. It frequently spreads longitudinally along the bile duct wall and through perineural spaces, and it responds poorly to conventional systemic therapy (1, 2). For resectable pCCA, microscopically negative margins (R0 resection) remain a key determinant of long-term outcome (3). In contrast, R1 resection is associated with higher local recurrence and inferior survival (3, 4). Accurate intraoperative assessment of the longitudinal bile duct margins is therefore central to surgical planning in pCCA.

Intraoperative frozen-section (FS) analysis of bile duct margins is a standard real-time method for guiding the extent of resection, but its diagnostic performance may be compromised by freezing artifacts, limited sampling depth, and the subtle cytologic atypia of well-differentiated adenocarcinoma (5, 6). Preoperative biliary drainage, including PTBD and endoscopic drainage, may further complicate interpretation by causing edema, inflammation, epithelial regeneration, and reactive atypia that can overlap morphologically with early malignant infiltration (7, 8). Direct quantitative data isolating the effect of PTBD on FS accuracy in pCCA remain limited; therefore, this case is best interpreted as a representative high-risk diagnostic scenario rather than as evidence of a drainage-modality-specific failure. We present a case in which distal-margin FS revealed only a few atypical glands of indeterminate nature, whereas permanent sections and immunohistochemistry confirmed margin involvement by well-differentiated adenocarcinoma. The aim of this report is to clarify how a gray-zone FS result can influence intraoperative surgical decisions, particularly when the patient's physiological reserve limits aggressive resection.

## Case description

**Figure 1:**
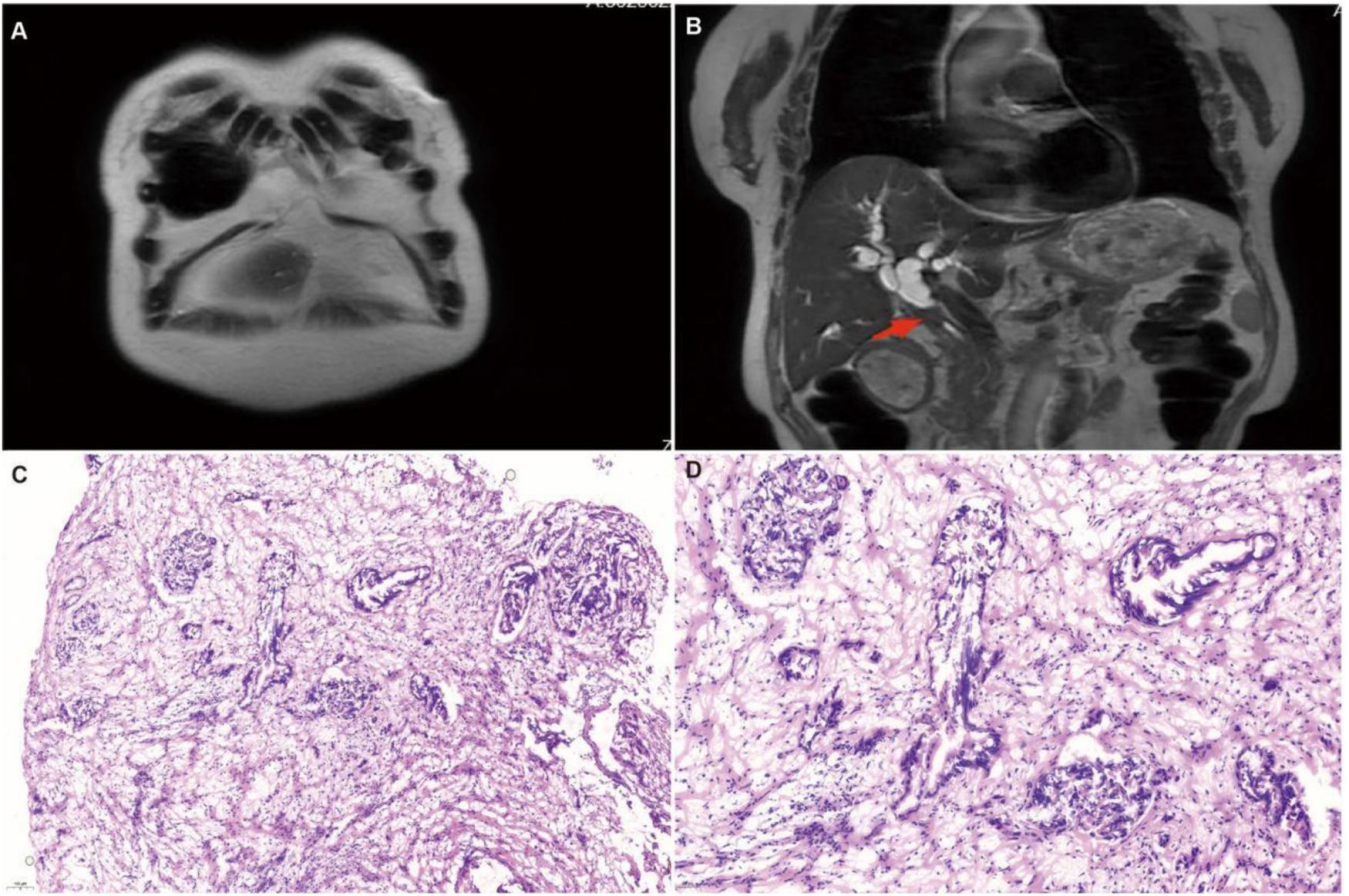
Imaging findings and intraoperative frozen-section HE staining of upper common bile duct carcinoma. **(A)** Axial T2-weighted upper abdominal MRI showing the lesion at the upper common bile duct. **(B)** Coronal T2-weighted upper abdominal MRI showing intrahepatic bile duct dilatation and an obstructive lesion in the upper common bile duct (red arrows). **(C)** Hematoxylin and eosin staining of the intraoperative frozen section (100×). **(D)** Hematoxylin and eosin staining of the intraoperative frozen section (200×).

A 70-year-old woman presented with a 3-day history of progressive jaundice involving the skin and sclera, accompanied by fatigue and an unintentional 3.5-kg weight loss over the preceding 2 months. Physical examination showed marked jaundice and scleral icterus, without palpable abdominal masses, hepatosplenomegaly, or peritoneal irritation. Laboratory tests demonstrated severe obstructive jaundice: total bilirubin 187.0 umol/L, direct bilirubin 113.1 umol/L, and gamma-glutamyl transferase 1,474 U/L. Contrast-enhanced CT and MRCP showed focal wall thickening with linear enhancement in the upper common bile duct, measuring approximately 13.8 mm in length. The proximal intrahepatic and extrahepatic bile ducts were markedly dilated, whereas the distal common bile duct was not dilated. An atrophic gallbladder with inspissated bile was also observed (Figures 1A,B). The working diagnosis was Bismuth-Corlette type II pCCA with obstructive jaundice. To improve hepatic function and reduce operative risk, PTBD was performed 16 days before definitive surgery, followed by gradual bilirubin reduction. Her relevant background included well-controlled hypertension treated with amlodipine, no history of diabetes mellitus, coronary heart disease, viral hepatitis, smoking, or alcohol use, and knee arthroplasty 5 months before admission. These factors, together with her age and severe obstructive jaundice, were considered during preoperative surgical-risk discussion. The clinical course and key decision points are summarized in Table 1.

**Table 1:** Clinical timeline and decision-relevant factors in the present case.

Time point	Clinical/pathological event	Decision relevance
Admission	Severe obstructive jaundice: total bilirubin 187.0 μmol/L, direct bilirubin 113.1 μmol/L, γ-GT 1474 U/L	High perioperative risk and need for biliary decompression before definitive surgery
16 days before surgery	PTBD performed	Improved biliary obstruction but created possible drainage-related epithelial/inflammatory confounding
Operation	Extrahepatic bile duct resection, lymphadenectomy, cholecystectomy, Roux-en-Y hepaticojejunostomy	Standard radical operation planned for suspected Bismuth-Corlette type II pCCA
First FS	Distal bile duct margin: a few atypical glands of undetermined nature	High-risk gray-zone result; malignancy could not be excluded
Second FS	Left and right proximal margins negative for malignancy	No proximal extension performed
Permanent pathology	Well-differentiated cholangiocarcinoma involving distal margin; perineural invasion; lymph-node metastasis 1/12	Confirmed unintended R1 resection and need for adjuvant therapy plus intensive surveillance

## Diagnostic assessment

The preoperative differential diagnosis primarily included perihilar cholangiocarcinoma, IgG4-related sclerosing cholangitis, and benign biliary strictures, with imaging strongly favoring a malignant etiology. Intraoperative FS evaluation of the bile duct margins was routinely performed in two separate submissions to guide the extent of resection. The distal bile duct margin was submitted first; microscopic examination revealed scattered glandular structures in the submucosa, accompanied by mild nuclear enlargement and atypia (Figures 1C,D). However, obscured by tissue edema secondary to the prior PTBD and inherent freezing artifacts, the pathologist struggled to definitively distinguish reactive biliary hyperplasia from well-differentiated adenocarcinoma infiltration. Consequently, the FS report concluded: A few atypical glands of undetermined nature; permanent sections recommended for definitive diagnosis. Subsequent FS evaluations of the proximal margins (left and right hepatic ducts) were negative for malignancy. At our institution, intraoperative FS assessment of bile duct margins is a mandatory step during surgery for suspected pCCA; however, at the time of this operation there was no formal written protocol specifically defining management of an “indeterminate” FS margin. In this case, the finding was discussed in real time between the operating hepatobiliary surgeons and the pathologists, and the final intraoperative decision incorporated the FS wording, macroscopic tumor boundaries, the absence of obvious vascular invasion, the patient's age, controlled comorbidity profile, and recent severe jaundice.

**Figure 2:**
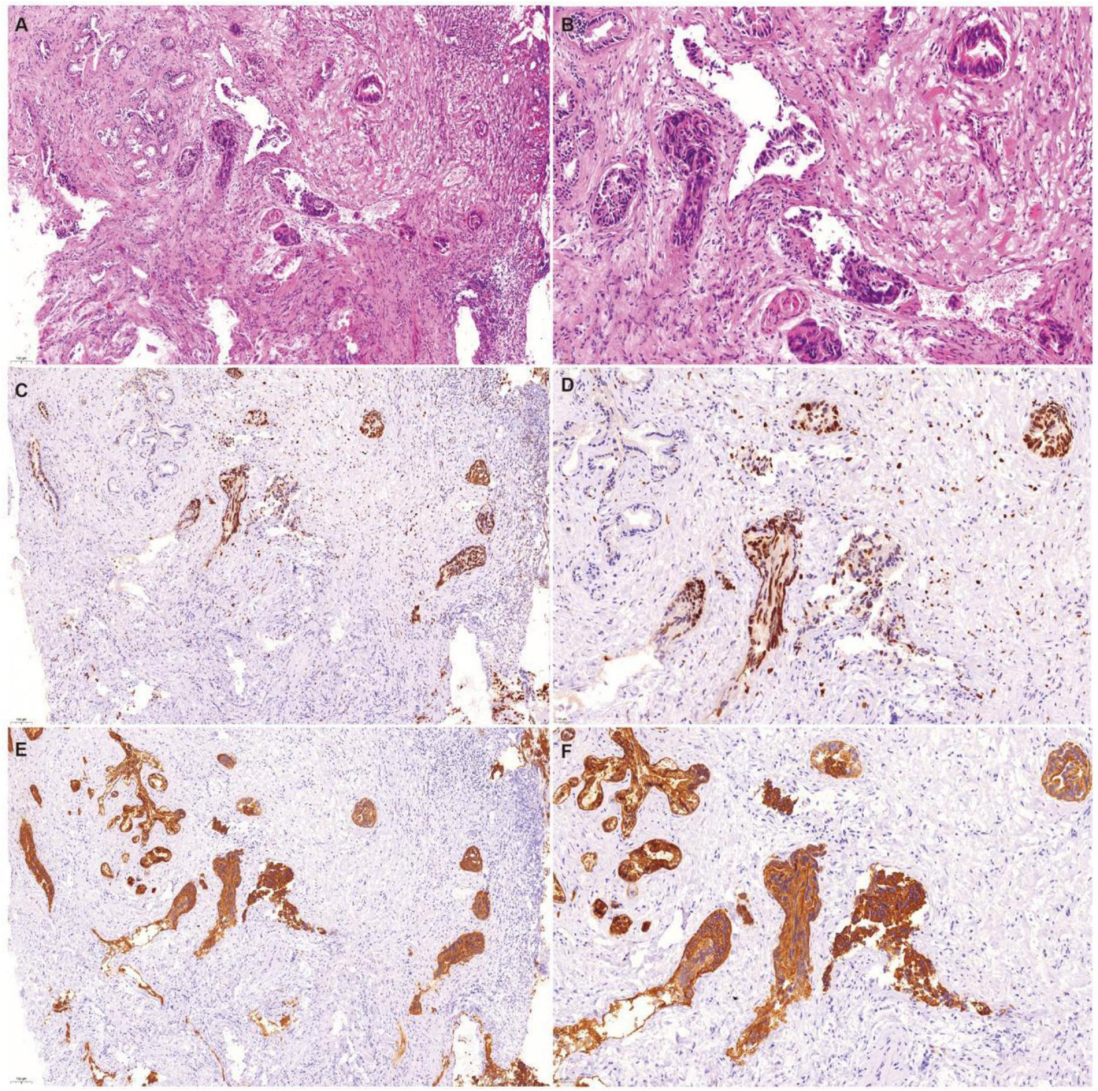
Histopathology and immunohistochemical staining of adenocarcinoma of the upper common bile duct. **(A,B)** Hematoxylin and eosin staining of permanent paraffin sections after intraoperative frozen examination; **(A)** (100×), **(B)** (200×). **(C,D)** Ki-67 immunohistochemical staining; **(C)** (100×), **(D)** (200×). **(E,F)** CK8 immunohistochemical staining; **(E)** (100×), **(F)** (200×).

Postoperative permanent sections and immunohistochemistry resolved the intraoperative uncertainty. Histological examination demonstrated well-differentiated adenocarcinoma infiltrating the submucosa of the distal bile duct margin, with prominent perineural invasion (Figures 2A,B). Immunohistochemistry showed strong CK8 expression in tumor cells and a Ki-67 expression higher than that of adjacent non-neoplastic biliary epithelium (Figures 2C,D). The final diagnosis was well-differentiated cholangiocarcinoma involving the distal resection margin (R1 resection), with regional lymph-node metastasis (1/12). These findings confirmed that the indeterminate FS margin represented malignant involvement rather than reactive atypia alone.

### Therapeutic intervention, follow-up and outcomes

The patient underwent radical resection for pCCA, comprising extrahepatic bile duct resection, regional lymphadenectomy, cholecystectomy, and Roux-en-Y hepaticojejunostomy. The indeterminate distal-margin FS report placed the surgical team in a difficult clinical dilemma. Achieving an unequivocally negative distal margin would have required more caudal resection and could have necessitated combined pancreaticoduodenectomy (PD). In a 70-year-old patient who had recently presented with severe obstructive jaundice, this extension was judged to carry substantially increased perioperative risk. The decision not to proceed to PD was therefore not regarded as a technical error, but as a complex trade-off between oncologic radicality and physiological tolerance. In retrospect, the decision process was vulnerable to ambiguity aversion, risk aversion, and status quo bias: the lack of a definitive malignant FS diagnosis made continuation of the planned operation appear safer than escalation to a more morbid procedure.

Postoperatively, the patient recovered well without major surgical complications or biliary leakage. Given the high recurrence risk associated with an R1 margin and lymph-node positivity, she received adjuvant gemcitabine plus cisplatin rather than the lower-risk postoperative pathway generally considered for patients with R0/N0 disease at our institution. At the 1-year postoperative follow-up, she remained asymptomatic and independently ambulatory. Tumor markers (CA19-9, CEA, and CA125), liver and renal function tests, and electrolyte levels were within normal limits, and contrast-enhanced abdominal CT showed no evidence of local recurrence or distant metastasis. The patient has been enrolled in a 5-year surveillance protocol: clinical review, liver biochemistry, and tumor markers every 3 months during the first 2 years; contrast-enhanced CT or MRI/MRCP every 3–6 months during the first 2 years; follow-up every 6 months during years 3–5; and annual surveillance thereafter or earlier imaging if symptoms, cholestasis, or marker elevation occurs.

## Discussion

In the surgical management of pCCA, achieving an R0 resection remains the cornerstone for improving long-term survival (9). FS analysis serves as the standard modality for real-time margin assessment, with its findings directly influencing the final extent of resection. The diagnostic dilemma in this case likely reflected several overlapping factors: well-differentiated tumor morphology, the small and thin-walled bile duct margin specimen, freezing-related loss of nuclear detail, and drainage-related epithelial reaction. PTBD can reliably decompress obstructed bile ducts, but any biliary drainage procedure, including ENBD, may be accompanied by cholangitis, epithelial regeneration, and inflammatory atypia (10, 11); thus, the key practical issue is not simply the choice of drainage route, but recognition that previously instrumented bile ducts are difficult FS specimens. Although published data specifically quantifying PTBD-induced reductions in FS sensitivity in pCCA are scarce, studies of pCCA margin status and bile duct pathology consistently emphasize that FS results must be interpreted together with clinical, radiologic, and macroscopic information (4–6).

Traditional surgical protocols often treat a definitively positive margin as the clearest indication for extended resection, whereas no universally accepted strategy exists for indeterminate gray-zone margins. This case should not be interpreted as a rare curiosity. In routine hepatobiliary practice, pathologists may avoid overcalling carcinoma on FS when tissue quality is suboptimal, while surgeons must make irreversible decisions under time pressure. The dangerous cognitive step is to convert “malignancy cannot be excluded” into “margin is probably safe.” In this patient, that step was reinforced by risk aversion toward PD in an elderly patient with recent obstructive jaundice, ambiguity aversion toward a non-binary pathology report, and status quo bias favoring completion of the originally planned operation. Permanent sections subsequently confirmed distal margin carcinoma and perineural invasion, proving that the indeterminate FS result was an oncologic warning signal. Although no recurrence was observed at 1 year, R1 resection in pCCA carries an ongoing risk of late local recurrence; therefore, no durable oncologic conclusion can be drawn from this short follow-up interval.

The “presumed positive” principle should not be misunderstood as a mandate for automatic maximal resection in every patient. Rather, it should trigger a structured escalation pathway. When physiological reserve is adequate, the surgical team should submit additional oriented distal tissue, request deeper or repeat FS levels when feasible, consider circumferential mapping of the bile duct margin, and proceed with extended resection only if the incremental oncologic benefit justifies the operative risk. When aggressive resection is physiologically contraindicated, as in elderly patients recovering from severe jaundice or those with poor cardiopulmonary reserve, priority should shift to immediate intraoperative MDT consultation, explicit documentation that malignancy cannot be excluded, avoidance of falsely reassuring terminology, early postoperative confirmation by permanent sections and immunohistochemistry, timely adjuvant therapy, and intensified surveillance. Neoadjuvant therapy is not a practical intraoperative rescue strategy once an unexpected gray-zone margin has been encountered, but it should be discussed preoperatively in future patients with borderline resectability or high anticipated risk of R1 resection.

This case prompted us to refine our institutional workflow for indeterminate FS margins in pCCA. We now distinguish three intraoperative categories: negative, indeterminate/high-risk, and positive (Table 2). For indeterminate results, the pathologist is asked to communicate the specific reason for uncertainty, the surgeon reorients and resamples the margin whenever technically feasible, and the team documents the physiological reasons if extended resection is not performed. Potential adjuncts, including imprint cytology, additional level sections, rapid immunohistochemistry, molecular margin assessment, and intraoperative imaging, may help in selected centers, but most are limited by turnaround time, validation status, or availability. Therefore, the most immediately generalizable intervention is disciplined communication and predefined decision thresholds rather than reliance on a single new technology.

**Table 2:** Proposed three-tier intraoperative margin risk-stratification framework for pCCA.

FS category	Operational interpretation	Recommended intraoperative response
Negative	No malignant or suspicious glandular infiltration identified	Proceed with planned resection while integrating macroscopic findings and specimen orientation.
Indeterminate/high-risk	Atypical glands, suspicious but non-diagnostic changes, or uncertainty caused by artifacts/inflammation	Treat as presumed positive: immediate surgeon-pathologist communication, repeat oriented sampling, additional/deeper levels if feasible, MDT risk-benefit discussion, and explicit documentation if extension is not performed.
Positive	Definite carcinoma at the margin	Extend resection when physiologically tolerable; if contraindicated, document rationale and prioritize adjuvant therapy and intensified surveillance.

This report has limitations. It describes a single patient, and our institutional experience was not analyzed as a formal case series; therefore, the frequency of this scenario cannot be estimated from the present report. In addition, the 1-year disease-free interval is reassuring but insufficient to draw long-term oncologic conclusions after R1 resection. These limitations are the reason we emphasize a surveillance protocol rather than presenting the short follow-up as evidence of durable disease control.

In summary, intraoperative indeterminate FS margins in pCCA should be treated as high-risk oncologic signals. A structured algorithm can reduce decision-making bias, but the final operative strategy must remain individualized to the patient's physiological reserve. When R0 resection cannot be safely achieved, transparent documentation, adjuvant therapy, and prolonged surveillance become integral components of care.

## Patient perspective

The patient reported complete resolution of obstructive jaundice and fatigue after surgery, with no new postoperative symptoms. She was informed that the final pathology showed an R1 resection and regional lymph-node metastasis, and that these findings require long-term surveillance beyond the currently disease-free 1-year interval. She understood the reason for adjuvant chemotherapy and agreed to comply with the planned 5-year imaging and tumor-marker follow-up schedule.

## Data Availability

The original contributions presented in the study are included in the article/Supplementary Material, further inquiries can be directed to the corresponding authors.
